# The Impact of Post-Stroke Disability on Rehabilitation Costs in Romania

**DOI:** 10.3390/jcm14228014

**Published:** 2025-11-12

**Authors:** Adriana Maria Canciu, Alina Liliana Pintea, Cosmina Diaconu, Florina Ligia Popa, Horațiu Paul Domnariu, Carmen Daniela Domnariu

**Affiliations:** 1 Faculty of Medicine, “Lucian Blaga” University of Sibiu, 550024 Sibiu, Romania; alina-liliana.pintea@ulbsibiu.ro (A.L.P.); cosmina.diaconu@ulbsibiu.ro (C.D.); florina-ligia.popa@ulbsibiu.ro (F.L.P.); horatiupaul.domnariu@ulbsibiu.ro (H.P.D.); carmen.domnariu@ulbsibiu.ro (C.D.D.); 2 County Clinical Emergency Hospital of Sibiu, 550245 Sibiu, Romania

**Keywords:** post-stroke disability costs, disability post-stroke, rehabilitation costs, direct costs, stroke survivors, disability assessment, Romania

## Abstract

**Background/Objectives**: Post-stroke disability is a prevalent complication in patients who have experienced a stroke. It is imperative that patients suffering from associated disability be hospitalised in rehabilitation wards, with a view to minimising their disability. The primary objective of the present study is to analyse the direct medical costs associated with the rehabilitation of patients with post-stroke disability who are admitted to a rehabilitation clinic for the first time. **Methods**: This retrospective study was conducted in a public hospital in Romania between January 2021 and December 2024. Patient information was retrieved from the hospital database and included the following: socio-demographic and clinical characteristics; disability score assessed using the modified Rankin scale (mRS); number of days of hospitalisation; and direct medical costs related to hospitalisation. **Results**: A total of 584 patients were included in this study. The average age was 68.04 years, 82% had suffered an ischaemic stroke, and 18% had suffered a haemorrhagic stroke. The mRS disability scores for ischaemic stroke were 2 (28.54%); 3 (24.79%); 4 (30.41%); and 5 (16.25%). The mRS scores for haemorrhagic stroke were 4 (33.65%); 5 (29.80%); 3 (20.19%); and 2 (16.34%). Hypertension was present in 80% of patients. The average length of hospital stay was 12.44 days. The total cost of hospitalisation per patient averaged RON 5295.33 thousand (approximately EUR 1031). Pearson’s correlation indicates a statistically significant positive association between higher mRS disability scores and higher hospitalisation costs (*p* < 0.001). **Conclusions**: The financial burden imposed on healthcare systems in Romania by medical expenses related to the rehabilitation of patients with post-stroke disability is significant. It is imperative to implement measures that will reduce the financial burden associated with hospitalising these patients and minimise the duration of their hospital stay.

## 1. Introduction

Stroke is a significant cause of long-term disability and is regarded as one of the most devastating neurological conditions. The condition affects approximately 14 million people worldwide each year [1,2].

The condition arises from cerebral impairment consequent to ischaemia (occlusion of a blood vessel) or haemorrhage (rupture of a blood vessel). Damage to the central nervous system has been demonstrated to compromise motor and cognitive functions [3,4].

It has been established that between 70 and 85% of stroke patients exhibit a motor deficit [5]. The resulting disability is a significant and long-term problem for the individual, their family, and society [6]. Following a stroke, approximately two-thirds of surviving patients continue to experience disability 15 years later [7].

In the context of an ageing population and an increase in the number of stroke survivors due to improved acute treatment, a corresponding increase in patients requiring long-term rehabilitation is anticipated. This will exert significant pressure on health budgets worldwide [8,9,10].

Romania has one of the highest stroke incidence rates in Europe, which has led to the identification of this as a significant public health concern on a national scale [11]. This phenomenon can be attributed to the high prevalence of stroke risk factors, including obesity, alcohol and tobacco use, and a sedentary lifestyle [12]. Additionally, the ageing population is a contributing factor. In the context of stroke treatment, medical facilities within our nation utilise therapeutic interventions such as intravenous thrombolysis and mechanical thrombectomy in suitable patients, with the objective of enhancing post-stroke functional outcomes [13]. Following the discharge of patients from a neurology ward, those afflicted with disabilities resulting from cerebrovascular accidents must be admitted to a rehabilitation clinic in order to commence specialised treatment aimed at minimising disability. Adherence to a rehabilitation programme has been demonstrated to reduce the societal burden of stroke [14]. Romania is aligning itself with other Eastern European countries, such as the Czech Republic and Poland, in terms of discharging patients from the neurology ward and admitting them to a rehabilitation department for early rehabilitation [15].

However, the number of neurological rehabilitation centres in the country is limited, with only a few offering admission for recovery treatment [11].

In relation to the financial implications of rehabilitation for patients with post-stroke disability who are hospitalised, it should be noted that the costs incurred vary between different geographical regions. For instance, in Poland, the associated costs are significantly lower than those documented in the United States [16,17].

Further research is required to determine the impact of disability on the costs associated with the rehabilitation of patients with post-stroke disability.

In this retrospective study, it was hypothesised that the financial burden of hospitalising patients with post-stroke disability in Romania is significant and that disability can substantially influence these costs. The objective of this study was twofold: firstly, to analyse the costs associated with hospitalising patients with post-stroke disability who were admitted to a rehabilitation clinic, and secondly, to explore the impact of post-stroke disability on direct medical costs.

## 2. Materials and Methods

### 2.1. Study Design

A retrospective monocentric study was conducted at Sibiu County Emergency Clinical Hospital, covering the period from 1 January 2021 to 31 December 2024. The main objective of this study was to analyse the costs of hospitalisation for patients with post-stroke disability who are admitted for the first time to a medical rehabilitation clinic in Romania.

The present study was approved by the Ethics Committee of the Sibiu County Emergency Clinical Hospital (No. 14663/12 June 2024) and the “Lucian Blaga” University of Sibiu (No. 28/25 September 2024). The present study is in accordance with the ethical principles set out in the Declaration of Helsinki.

### 2.2. Subjects and Data Source

The patients included in the present study had a history of either ischemic or haemorrhagic stroke and were admitted to a rehabilitation clinic within a public hospital in Romania.

The inclusion criteria for our study were as follows:

(a) Patients over 18 years of age;
(b) Patients with a history of stroke;
(c) Patients whose degree of disability was measured using a standardised instrument;
(d) Patients with no other causes of disability;
(e) Patients with stroke admitted to a rehabilitation clinic for the first time.

The exclusion criteria for the study were as follows:

(a) Patients under the age of 18.
(b) Patients who had not suffered a stroke in the past.
(c) Patients with concomitant neurological, orthopaedic, or rheumatological disabilities.

The following variables were extracted for each patient:

- Socio-demographic data (gender, age at admission, place of residence);
- Clinical characteristics: type of stroke and associated comorbidities according to ICD-10 coding, disability score;
- Number of days of hospitalisation;
- Medical costs related to accommodation, food, medication, medical supplies, and laboratory tests.

The data were obtained from the database of the Sibiu County Emergency Clinical Hospital. The cases were identified in the hospital database using the specific stroke diagnosis codes between I60.0, I60.1, I60.2, I60.3, I60.4, I60.5, I60.6, I60.7, I60.8, I63.0, I63.1, I63.2, I63.3, I63.4, I63.5, I63.6, I63.7, I63.8, I63.9, and I69.0, I69.1, I69.2, I69.3, I69.4, I69.5, I69.6, I69.7, and I69.8.

It is imperative to note that the identification data of the study cohort were removed prior to the processing stage.

The assessment of disability was conducted using the Modified Rankin Scale (mRS). This scale is a tool for measuring post-stroke disability that is frequently utilised in clinical studies. The scale in question comprises seven distinct levels, with the mRS score representing the following: 0—no symptoms present; 1—no significant disability; 2—mild disability; 3—moderate disability; 4—moderate-severe disability; 5—severe disability; and 6—deceased [18].

### 2.3. Statistics

The database we used was created using internal hospital data on patient history and was imported in Microsoft Excel (Redomond, WA, USA). The data was preprocessed using the “tidyverse” data mining and processing library from the R project for statistical computing (v4.5.1). The data was further analysed using JASP Software v.0.19.3—company name JASP Services BV (Adresse Company Software, Amsterdam, The Netherlands). We used frequency and percentages to present categorical data, and for continuous data we computed standard deviation—SD, and mean—M. The tests we conducted used inferential statistics for their base, where we tested first for normality assumptions with Shapiro–Wilk, and then with Mann–Whitney U, or student tests, depending on the results. Further, we used linear regressions, chi-square tests, and Pearson and Spearman tests, depending on the variable normal distribution. We used a 0.05 threshold for determining statistical significance.

### 2.4. Calculation of Cost

The total amount of costs incurred by a person suffering from post-stroke disabilities and requiring hospitalisation can be classified as follows:

- Accommodation costs;
- Food costs;
- Medication costs;
- Medical supplies costs;
- Laboratory analyses costs.

The categorisation of costs is standardised by Romanian healthcare services, with costs allocated as fixed expenses predetermined per day of hospitalisation.

In order to ascertain the total cost of hospitalisation, it is necessary to multiply the individual expenses related to accommodation, food, medication, laboratory tests, and medical supplies by the total number of days of hospitalisation.

## 3. Results

### 3.1. Assessment of Disability Following a Stroke

The study cohort comprised 584 patients who had suffered a stroke between 2021 and 2024 (see Table 1).

**Figure 1:**
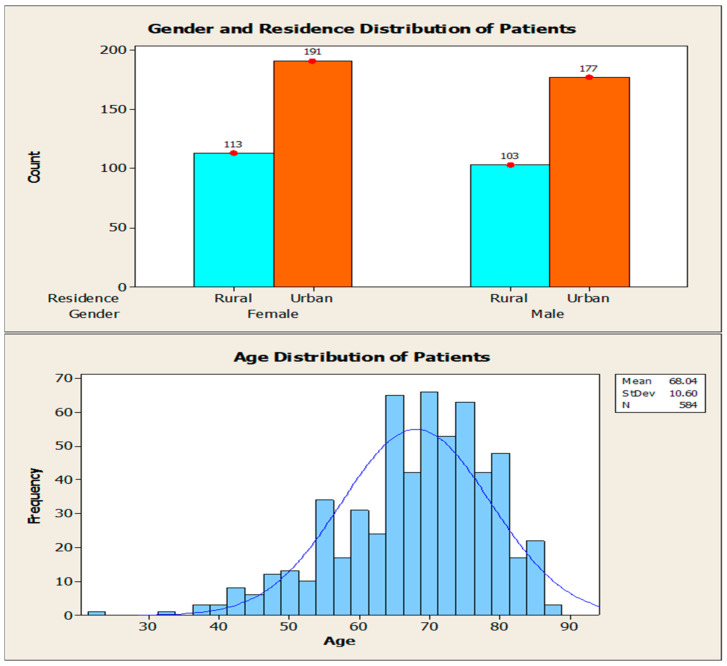
Socio-demographic characteristics of the cohort.

With regard to the socio-demographic data of the patients enrolled in this study, as illustrated in Figure 1, there was a slight predominance of females (n = 304, 52%) in comparison to males (n = 280, 48%). Geographically, the majority of patients were from urban areas (n = 368, 63%) compared to rural areas (n = 216, 37%). The mean age of the subjects was 68.04 years.

The clinical characteristics of the patients enrolled in this study are shown in Table 2. Ischemic stroke was predominant in these patients, accounting for 82% of cases (n = 480). Conversely, the prevalence of haemorrhagic stroke was found to be significantly lower, with a proportion of 18% (n = 104).

Hypertension was identified in 80% (n = 470) of the cohort, followed by ischemic coronary artery disease, mixed dyslipidemia, depression, diabetes mellitus, atrial fibrillation, and thrombophilia.

**Figure 2:**
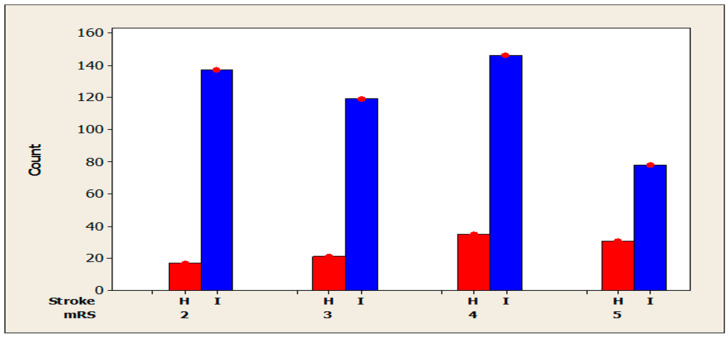
Disability score according to stroke type (H = Haemorrhagic; I = Ischemic).

The assessment of post-stroke disability for the study sample (n = 584) was performed using the mRS scale and is shown graphically in Figure 2. A descriptive analysis of the mRS variable indicated a mean of 3.42 (SD = 1.07), with values ranging from 2 to 5 (n = 584, no missing data). The Mann–Whitney U test demonstrated statistical significance (*p* < 0.001) in terms of disability level, thereby highlighting the existence of disparities between the two categories of stroke. The mRS scale indicates a higher mean (3.76, SD = 1.05) among patients with haemorrhagic stroke, and a lower mean (3.34, SD = 1.06) among patients with ischaemic stroke.

The chi-square test indicated a statistically significant association between the presence of mRS and stroke, X2 = 14.28, *p* = 0.003, (N = 584). The mRS distribution differs significantly between patients with haemorrhagic stroke and those with ischaemic stroke. A subsequent analysis of the distributions indicates that patients diagnosed with haemorrhagic stroke are more likely to exhibit elevated levels of disability. The proportion of patients with mRS scores of 4 or 5 was significantly higher in the case of haemorrhagic stroke (33.65% and 29.80%, respectively) compared to ischemic stroke (30.41% and 16.25%, respectively). Conversely, lower scores (2 or 3) are more prevalent in patients with ischemic stroke (28.54% for score 2 in ischemic stroke, compared to 16.4% in haemorrhagic stroke). In a similar vein, an incidence of score 3 in the context of ischemic stroke (24.79%) stands in contrast to the 20.19% observed in cases of haemorrhagic stroke.

The factors that may influence disability are shown in Table 3. In order to assess differences in mRS scores by gender, the Mann–Whitney U test was applied. The statistical analysis of the results revealed no statistically significant differences between the groups (*p* = 0.87, <0.05), suggesting that the distribution of mRS scores is comparable between male and female subjects. The differences in mRS between urban and rural patients were then analysed using the Student’s *T*-Test. In relation to the geographical location of residence, a substantial variation in mRS scores is observed between patients from urban and rural areas (*p* = 0.02, <0.05). The simple linear regression model indicated a statistically significant relationship between age and mRS (*p* < 0.01, *p* = 0.001). The Shapiro–Wilk normality tests were significant for both variables (MRS scale and age), indicating non-normal distributions. Therefore, the non-parametric Spearman correlation was used instead of the Pearson correlation to ensure robustness against violations of normality.

The coefficient of determination (β) was found to be 0.015 (SE = 0.004), indicating that for every year of age added, there is an average increase of 0.015 units in the score. In order to examine the association between the number of comorbidities and the degree of disability (mRS), Pearson’s correlation was used, which indicated a statistically significant positive association, albeit weak (r = 0.087, *p* = 0.035, *p* < 0.05, N = 584), showing that patients with more comorbidities had a higher score on the mRS scale.

### 3.2. Assessment of Inpatient Costs Related to Rehabilitation in Relation to Disability

**Figure 3:**
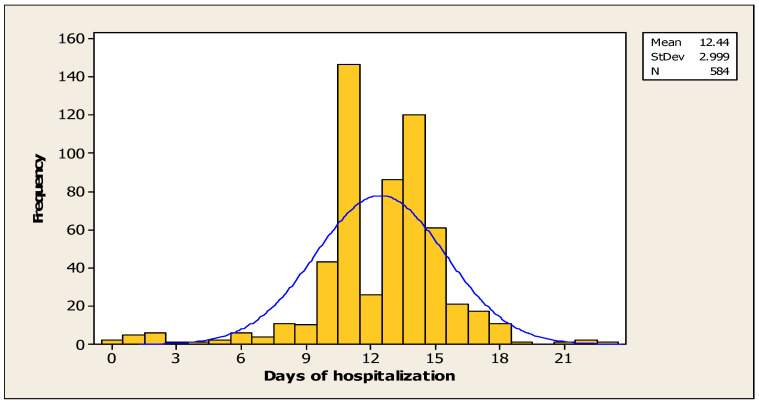
Number of days of hospitalisation for the cohort.

The average length of hospital stay for patients with post-stroke disability was 12.44 days (Figure 3).

In order to examine the association between the number of days of hospitalisation and the degree of disability (mRS), Pearson’s correlation was used, which indicated a statistically significant positive association, albeit a weak one (r = 0.110, *p* < 0.007). This suggests that patients with a higher degree of disability were hospitalised for a longer period of time.

For a sample of 584 patients with no missing data, the total average cost of hospitalisation was RON 5282 thousand (approximately EUR 1037), with a standard deviation of RON 1434.33 thousand (app. EUR 281), indicating moderate variability around the mean. The minimum reported value was RON 476,100 RON (app. EUR 93), while the maximum value reached RON 16,116,820 (app. EUR 3166).

**Figure 4:**
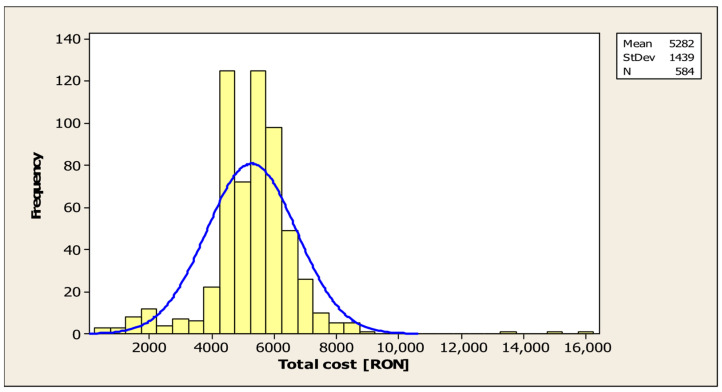
Average cost of hospitalisation for patients with post-stroke disability.

The density distribution exhibits a concentrated profile centred around the range of RON 4000–6000 thousand RON (app. EUR 786–1179), accompanied by a rightward asymmetry (positive), indicative of the presence of extreme but infrequent values with regard to costs. While the majority of cases are concentrated within a narrow range, a significant proportion of cases exhibit values in excess of RON 10,000 thousand (app. EUR 1965). This observation indicates a heightened severity of these cases, as illustrated in Figure 4.

**Figure 5:**
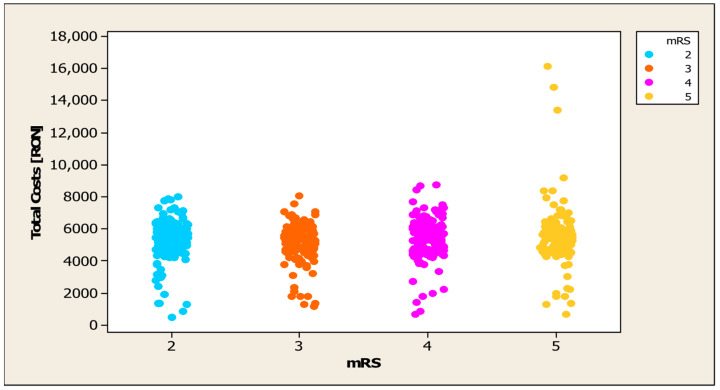
Distribution of mRS in relation to hospitalisation costs.

In order to examine the association between total expenditure and disability level (mRS), Pearson’s correlation coefficient was utilised, which indicated a statistically significant positive association (r = 0.147, *p* < 0.001). This indicates that patients with a higher degree of disability also incur higher hospitalisation costs (see Figure 5).

**Figure 6:**
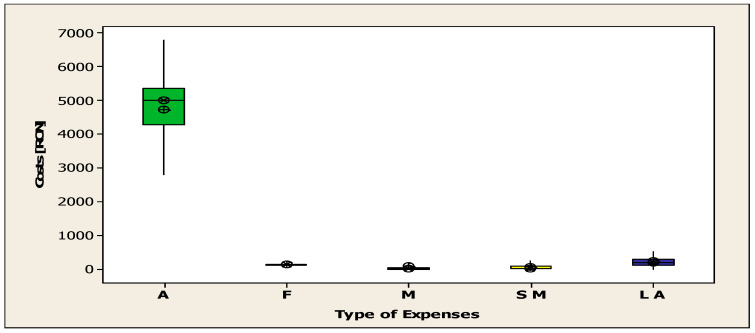
The average cost of accommodation, food, medication, sanitary materials, and laboratory tests (A = Accommodation; F = Food; M = Medication; SM = Sanitary Materials; LA = Laboratory Analyses).

The mean value of expenses is illustrated in Figure 6. The total expenditure on accommodation amounted to RON 4,756,190 (app. EUR 934) (SD = 1163.19), with a minimum of RON 230,580 (app. EUR 45) and a maximum of RON 8,508,000 (app. EUR 58) (SD = 1163.19). The minimum and maximum values were RON 230.58 thousand (app. EUR 45) and RON 8508.26 thousand (app. EUR 1671), respectively. The mean expenditure on food was RON 134,820 (approximately EUR 26) (SD = 30.13), with values ranging from RON 10,000 to 240,000 (approximately EUR 2–47). The mean expenditure on medicine was RON 94,630 (approximately EUR 118) (SD = 440.69), with a minimum of RON 0 and a maximum of RON 8864.48 thousand (approximately EUR 1741). With regard to medical supplies, the mean value was RON 70.07 thousand (approximately EUR 13) (SD = 98.76), with a range from ROM 0 to 1109.12 thousand (approximately EUR 217). For laboratory tests, the mean value was RON 239.73 thousand (approximately EUR 46) (SD = 211.56), with values ranging from RON 0 to 2897.00 thousand (approximately EUR 569).

## 4. Discussion

In this study, the primary objective was successfully achieved, demonstrating that the level of post-stroke disability exerts a significant influence on the direct costs of hospitalisation for rehabilitation. Consequently, an elevated disability score on the mRS scale is associated with increased costs of hospitalisation. Concurrently, several factors were identified that are associated with a higher disability score.

In terms of the distribution of patients by year, there was an increase in the number of hospitalised cases. The lowest number of hospitalised patients was in 2021. This is due to the COVID-19 pandemic, which continued into 2021, a year in which, on the one hand, measures to prevent the spread of the virus had to be observed and, implicitly, fewer patients were hospitalised, and on the other hand, patients avoided hospitalisation for conditions that were not life-threatening. Subsequently, there was a gradual increase in the number of patients. This increase in the number of hospitalisations is due to the fight against the COVID-19 pandemic and the increase in the number of stroke survivors who required neuromotor recovery.

The proportion of the cohort by gender was relatively balanced, with a slight predominance of females. This finding aligns with the conclusions of other studies, which suggest that the impact of stroke on women differs from that on men. As indicated by the extant literature, women who have suffered a stroke are more likely to survive this event, but have a higher level of disability compared to men [19]. However, the results of our study demonstrate that the mRS distribution is comparable between men and women. The majority of patients are from urban areas, with the remainder from rural areas. It is a well-documented fact that individuals residing in rural areas tend to benefit to a lesser extent from rehabilitation services when compared with their urban counterparts [20]. The mean age of subjects was 68.04 years, a figure that aligns closely with the findings of related studies [21].

With regard to the clinical characteristics of the patients, it was found that the vast majority of patients included in this study had a history of ischemic stroke, while the remainder had a history of haemorrhagic stroke. It is widely acknowledged that ischemic stroke represents the most prevalent form of stroke [22,23,24]. Hypertension was identified as the primary comorbidity in the study population [25]. This finding is consistent with data from the literature, which demonstrates that hypertension is the most common risk factor in both types of stroke [26]. The majority of patients included in the study exhibited an mRS score of 4, indicating moderate disability. A comparison of the two types of stroke reveals discrepancies in terms of the degree of disability exhibited. The mRS scale indicates a higher mean among patients with haemorrhagic stroke compared to ischemic stroke. This finding aligns with the conclusions drawn in other studies [27]. It has been demonstrated that haemorrhagic stroke is associated with a greater degree of disability in comparison with ischemic stroke. The rationale behind this phenomenon is that haemorrhagic strokes are more severe than ischaemic strokes due to the extent of the haemorrhage [28].

In addition to the type of stroke, it was demonstrated that age, place of residence, and number of associated pathologies are associated with a higher mRS score of [29]. With regard to age, patients of more advanced years have been shown to exhibit higher levels of disability. It is noteworthy that for each additional year of age, the disability score increases. These results contradict the data reported in the existing literature. A study conducted in 2011 in Turkey revealed that age constitutes a significant risk factor for stroke occurrence, as evidenced by a sample of 126 patients suffering from stroke who were treated in a rehabilitation clinic. However, the study concluded that age did not exert a substantial influence on the degree of disability [30].

The mean duration of hospitalisation for patients was 12.44 days. In comparison with other nations, the duration of hospitalisation for patients in this country is relatively brief. For instance, in Canada the average length of stay is 35 days [31], whereas in Arab countries the duration exceeds 40 days [32].

Patients exhibiting a higher level of disability required a longer period of hospitalisation. This may be attributable to the presence of severe motor deficits or the occurrence of potential complications that could have interfered with the recovery process. It is well established that stroke patients are predisposed to a heightened risk of complications, which have the potential to impede the process of rehabilitation [33].

The mean cost of hospitalisation for patients with post-stroke disabilities is analogous to the findings of other studies. Trung VQ et al. conducted a retrospective study similar to ours in Vietnam between 2014 and 2016, and demonstrated that the estimated rehabilitation costs are USD 985, which is consistent with the findings of our study [5]. A preliminary observation suggests that the expenses associated with initial admission to a rehabilitation clinic in our country are not excessively high in comparison to those observed in other countries [14,34]. On a global scale, the highest costs associated with the rehabilitation of patients with post-stroke disability were recorded in the US [17].

Patients with a more advanced level of disability require more days of hospitalisation to minimise motor deficits, generating higher hospitalisation costs. Our results are consistent with those of other studies. In 2017, Angerova Y. et al., following a study conducted in the Czech Republic, showed that total rehabilitation costs increase in direct proportion to the degree of disability [15].

The present analysis was conducted exclusively on the costs associated with the initial admission. Patients with a higher level of post-stroke disability may require multiple admissions to a rehabilitation clinic in order to minimise their disability, which can generate high costs.

The strengths of our study are as follows:

(a) To our knowledge, this is the first study in Romania to analyse the hospital costs associated with the rehabilitation of patients with post-stroke disability.
(b) Our research contributes to the field by comparing the costs associated with the rehabilitation of these patients and can be useful both in comparing our costs with those in other countries and in developing future studies related to disability.
(c) The objective of this study was to ascertain the financial implications of rehabilitation and post-stroke disability. To this end, patients with other causes of disability were excluded from this study. This potential ambiguity could have led to misinterpretation of the underlying causes of disability, potentially impacting the validity of our results.
(d) Another strength of this study is that the analysis of costs was based on disability, rather than on the type of stroke. As demonstrated, it is disability that exerts a modulating effect on costs, not the type of stroke.

Clinical implications of this study:

The results of this study underscore the importance of early assessment and stratification of post-stroke disability to optimise rehabilitation planning and resource allocation. By identifying patients with higher disability scores at an early stage, clinicians can implement individualised rehabilitation programmes that can reduce both the length of hospital stay and the total cost of care. Strengthening outpatient rehabilitation services could also reduce long-term costs and improve functional recovery and quality of life for patients.

Future research directions:

(a) Future research should focus on conducting prospective studies on a larger sample of patients, tracking rehabilitation costs over a longer period of time and including subsequent readmissions for patients requiring sustained rehabilitation treatment;
(b) Studies are also needed to evaluate the effectiveness of outpatient rehabilitation in patients with mild disabilities in order to reduce hospitalisation costs.

## 5. Limitations of This Study

It should be noted that our research has certain limitations, such as the following:

(a) Firstly, our study is retrospective, and was conducted in a single centre on a relatively small sample of patients, given its duration of 4 years. The small number of patients included is due to the fact that in 2021 there was the COVID-19 pandemic, and at that time it was preferred to avoid hospitalisations that were not medically urgent.
(b) Secondly, the analysis was only possible for expenses related to the initial admission. As this was a retrospective study, with data taken from the hospital database, it was quite difficult to track patients over time, which could lead to confusion. This confusion may be attributable to the fact that following their initial admission to our department, patients may continue their rehabilitation in other centres to which we do not have access to their database, thus preventing us from obtaining a comprehensive overview of the total cost of these expenses.

## 6. Conclusions

The financial burden imposed on healthcare systems in Romania by medical expenses related to the rehabilitation of patients with post-stroke disability is significant. To address this issue, it is imperative to implement measures that will reduce the financial burden associated with hospitalising these patients and minimise the duration of their hospital stay.

The enhancement of outpatient rehabilitation services has the potential to curtail these expenditures in the long term and enhance quality of life. Subsequent studies ought to examine the effect of referring patients with mild disabilities to outpatient rehabilitation, with the objective of decreasing these expenditures.

## Figures and Tables

**Table 1:** Distribution of hospitalised patients by year.

Year	Number of Patients	Percentage [%]
2021	90	15.41
2022	131	22.43
2023	178	30.48
2024	185	31.68
Total	584	100

**Table 2:** Clinical characteristics of patients.

Parameter		Study Group (n = 584)	
Type of Stroke	Ischemic	82% (n = 480)	
	Haemorrhagic	18% (n = 104)	
Type of Motor Deficit	Hemiparesis	49.66% (n = 290)	
	Hemiplegia	50.34% (n = 294)	
Associated Pathologies		Present	Absent
	Hypertension	80% (n = 470)	20%(n = 114)
	Coronary Artery Disease	21.64% (n = 148)	78.36% (n = 436)
	Mixed Dyslipidaemia	20.38% (n= 119)	79.62% (n = 465)
	Depression	9.76% (n = 57)	90.24% (n = 527)
	Diabetes mellitus	8.05% (n = 47)	91.95% (n = 537)
	Atrial Fibrillation	2.91% (n = 17)	97.09% (n = 567)
	Thrombophilia	2.57% (n = 15)	97.43% (n = 569)

**Table 3:** Factors that can influence disability.

Parameter	*p*-Value
Age	<0.001
Gender	0.87
Residence	0.02
Stroke type	<0.001
Number of Associated Pathologies	0.035

## Data Availability

The original contributions presented in this study are included in the article. Further inquiries can be directed to the corresponding authors.
